# The global risk of infectious disease emergence from giant land snail invasion and pet trade

**DOI:** 10.1186/s13071-023-06000-y

**Published:** 2023-10-17

**Authors:** Jérôme M W Gippet, Olivia K Bates, Jérémie Moulin, Cleo Bertelsmeier

**Affiliations:** 1 Department of Ecology and Evolution, University of Lausanne, 1015 Lausanne, Switzerland; 2 Association OPPAL - Chemin de la Cotze 26, 1941 Vollèges, Switzerland

**Keywords:** Biological invasions, Emerging disease, Exotic pets, Instagram, *Lissachatina fulica*, Social media, Zoonoses

## Abstract

**Background:**

Pathogen outbreaks mostly originate from animals, but some species are more likely to trigger epidemics. The giant land snail (*Lissachatina fulica*) is a widespread invader, a popular exotic pet, and a notorious vector of the rat lungworm, causing eosinophilic meningitis in humans. However, a comprehensive assessment of the risks of disease outbreak associated with this species is lacking.

**Methods:**

We assessed and mapped the risk of disease transmission associated with the invasion and pet trade of *L. fulica*. First, we conducted a review of the scientific literature to list all known *L. fulica* parasites and pathogens and query host–pathogen databases to identify their potential mammalian hosts. Then, to assess the potential for *L. fulica* to spread globally, we modelled its suitable climatic conditions and tested whether, within climatically suitable areas, the species tended to occur near humans or not. Finally, we used social media data to map *L. fulica* possession as an exotic pet and to identify human behaviours associated with increased risk of disease transmission.

**Results:**

*Lissachatina fulica* can carry at least 36 pathogen species, including two-thirds that can infect humans. The global invasion of *L. fulica* is climatically limited to tropical areas, but the species is strongly associated with densely populated areas where snails are more likely to enter in contact with humans. In temperate countries, however, climatic conditions should prevent *L. fulica*'s spread. However, we show that in Europe, giant snails are popular exotic pets and are often handled with direct skin contact, likely increasing the risk of pathogen transmission to their owners.

**Conclusions:**

It is urgent to raise public awareness of the health risks associated with *L. fulica* in both tropical countries and Europe and to regulate its trade and ownership internationally. Our results highlight the importance of accounting for multiple types of human-wildlife interactions when assessing risks of infectious disease emergence. Furthermore, by targeting the species most likely to spread pathogens, we show that it is possible to rapidly identify emerging disease risks on a global scale, thus guiding timely and appropriate responses.

**Graphical Abstract:**

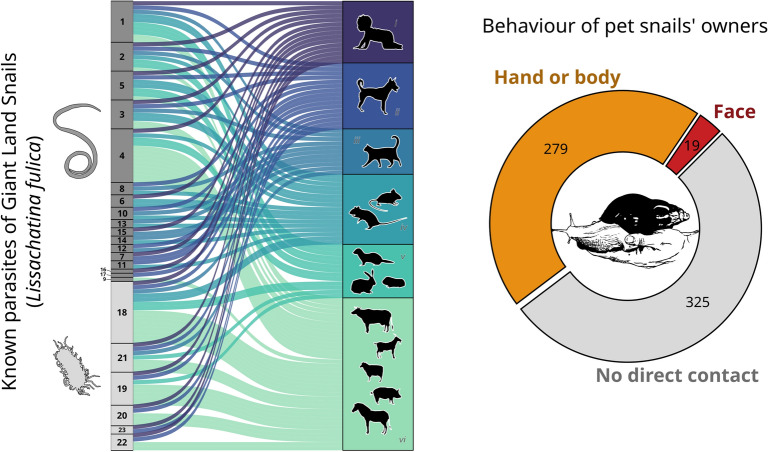

**Supplementary Information:**

The online version contains supplementary material available at 10.1186/s13071-023-06000-y.

## Background

Emerging infectious diseases are a major and growing threat to biodiversity and human societies worldwide. The emergence and spread of novel pathogens have already wiped out entire species, led to the death of millions of farmed animals and profoundly impacted humans throughout history [1–3]. Wild animals are the most frequent origin of such outbreaks [4]. However, some species are more likely to be the source of epidemics than others for three key reasons. First, species that are reservoirs for many or particularly virulent pathogens have a higher probability of spreading harmful diseases [5, 6]. Second, introduced species can spread new pathogens globally or propagate known diseases as they are abundant and widespread, especially in densely populated areas. This increases chances of spill-over to other animals and humans [7, 8]. Finally, species that are directly ingested by humans (as food or ingredients for traditional medicine) or kept as non-traditional pets are more likely to cause outbreaks due to frequent and close contact with humans [6]. It is therefore essential to assess public health risks associated with species that meet one or more of these criteria as they are the most likely sources of future epidemic events.

The giant land snail *Lissachatina fulica*, the largest terrestrial gastropod, meets all of these criteria: (i) the species is a vector of the rat lungworm, a parasitic nematode that can cause severe health impairments in humans [9]; (ii) it originates from East Africa and is currently spreading to other parts of the world with self-sustaining populations outside of its native range [10] (cabidigitallibrary.org); (iii) it can be easily purchased in physical and online stores as an exotic pet [11, 12]. Surprisingly, however, a comprehensive global assessment of the risk of zoonotic disease emergence associated with the giant land snail’s global invasion and pet trade is still lacking. Here, we assessed and mapped the risk of transmission of pathogens and parasites (hereafter ‘pathogens’) from giant land snails to humans and other mammals (wild and domestic) globally.

## Methods

### Pathogens carried by giant land snails and their potential mammalian hosts

**Fig. 1:**
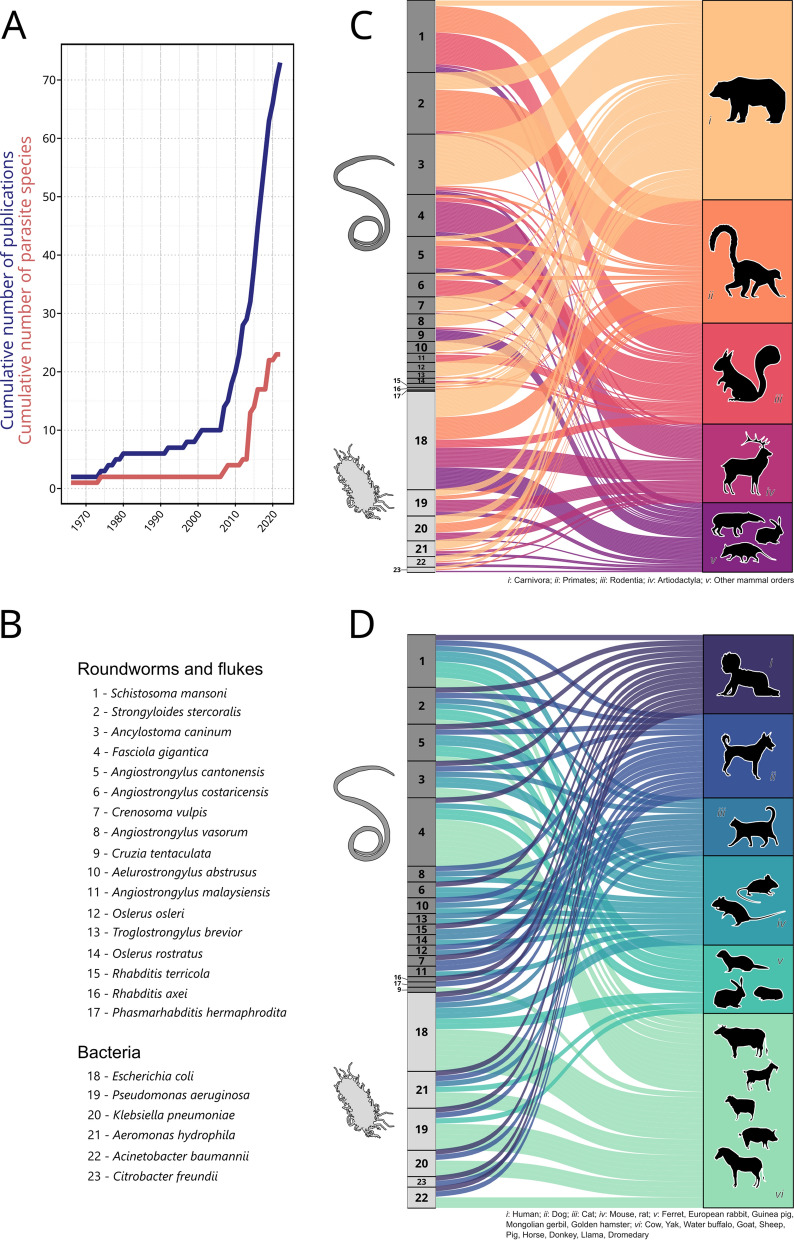
Pathogens carried by the giant land snail, *Lissachatina fulica*. **A** Cumulative number of scientific publications documenting pathogens in *L. fulica* and cumulative number of pathogen species (identified to the species level) documented in these publications. **B** List of the 17 helminths and six bacteria species identified with** C** their potential hosts among all mammals (*n* = 248 species) and **D** among humans and domesticated mammals (*n* = 19). Each curve in the alluvial plots represents a documented association between a pathogen and a host

To list all known parasites and pathogens that are carried by *L. fulica*, we performed a literature review on Web of Science (on October 4, 2022) with the query: (“lissachatina fulica” OR “achatina fulica”) AND (parasit* OR pathogen* OR zoonos* OR virus* OR bacteria* OR worm* OR helminth* OR fung*). We then reviewed the 192 papers found, excluded irrelevant papers based on title and abstract, and listed parasite and pathogen species associated with *L. fulica* by reading the abstract or the entire text of relevant papers (*n* = 73; see Supplementary information for details). Papers were only included if they reported a direct observation of an association between *L. fulica* and one or more parasite or pathogen species. This screening step was performed twice (by JMWG and JM, independently) to ensure that no host-pathogen interaction was omitted. We then determined which species can be infected by these pathogens by searching for host-parasite interactions in four extensive databases: the global biotic interactions database (GloBI) [13], London Natural History Museum’s host-parasite database (LNHM host-parasite database) [14], enhanced infectious disease database (EID2) [15] and global mammal parasite database version 2.0 (GMPD2) [16]. The GloBI and LNHM host-parasite databases were accessed on October 10, 2022, using their respective R packages [14, 17]. The EID2 and GMPD2 were accessed through the October 2020 release of the CLOVER database [18]. Host names were then checked for validity and synonymity and harmonized using the R package taxize [19]. Alluvial plots were used to visualize the host-pathogen interactions (Fig. 1C, D) with the geom_*alluvium* function of the ggalluvial R package [20].

### Environmental suitability for giant land snails

Global climatic suitability for *L. fulica* was computed using bioclimatic variables derived from monthly temperature and rainfall values [21] and represent annual trends (e.g. mean annual temperature, annual precipitation), seasonality (e.g. annual range in temperature and precipitation) and extreme or limiting environmental factors (e.g. temperatures of the coldest and warmest month, and precipitation of the wet and dry quarters). We used a spatial resolution of 2.5 arcmin (~ 5 km). These 19 variables were then reduced to 5 by conducting a principal components analysis (PCA) on the world maps using the function ‘PCAraster’ [22] in R to account for collinearity between the different variables (Additional file 1: Figs. S1, S2).

**Fig. 2:**
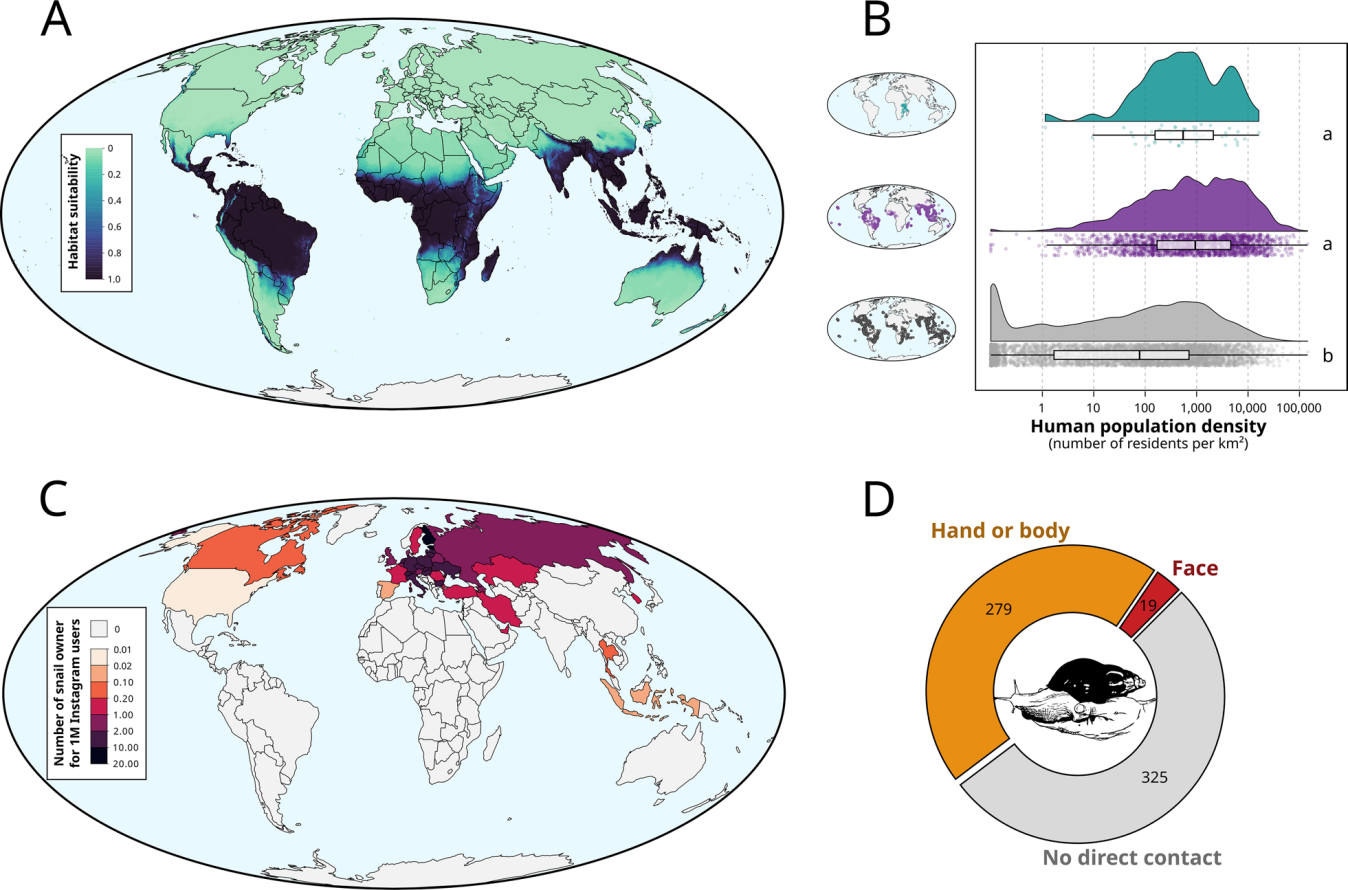
Geographical patterns of invasion risk **A**, **B** and pet trade **C**, **D** in giant land snail, *Lissachatina fulica*. **A** Forecast of global climatic suitability for *L. fulica* based on an ensemble of seven algorithms using 19 climatic variables reduced to five axes using a principal component analysis. Model’s performance was high (TSS = 0.87). **B** Human population density where *L. fulica* occurs (native and invasive range) compared to background occurrences (i.e. records of other terrestrial gastropods inside the climatically suitable area for *L. fulica*). Small maps depict the location of occurrences for each group (*L. fulica* native = 61, *L. fulica* invasive = 1480, background = 115,162). **C** Distribution of Instagram users posting about their pet giant land snails. **D** Proportion of Instagram users displaying direct skin contact with their pet snails

To calibrate and validate our models, we obtained > 11,000 native and invasive occurrences of *L. fulica* from GBIF (gbif.org; Fig. 2B). For background points (pseudo-absences), we used the > 3,000,000 occurrences of terrestrial gastropods (Stylommatophora) from GBIF [23]. All occurrences were then cleaned by excluding records with imprecise (i.e. > 1 km inaccuracy), invalid (i.e. equal latitude and longitude, coordinates equal to zero, coordinates outside land masses) or dubious coordinates (i.e. duplicated coordinates, coordinates corresponding to country centroid or capitals) using the CoordinateCleaner R package [24]. For each species, occurrences were thinned so that remaining occurrences were at least 5 km apart from each other using the R package ‘SpThin’ [25]. This was done to limit spatial autocorrelation biases that may arise if some locations are heavily sampled and that can affect the results of species distribution models [26]. The resulting data have 1541 occurrences for *L. fulica* and 115,162 background occurrences. We randomly subsampled 1541 background occurrences to ensure equal weighting of presence and background occurrences in our models. We produced 10 sets of subsampled background occurrences which were run separately for each model used (Additional file 1: Fig. S3).

To assess environmental suitability for *L. fulica* at global scale, we used an ensemble model by creating an ensemble prediction from the predictions of seven ecological niche models in the Biomod2 package [27]: (i) generalized linear model (GLM), (ii) generalized boosting model (GBM), (iii) classification tree analysis (CTA), (iv) artificial neural networks (ANN), (v) multiple adaptive regression splines (MARS), (vi) random forest (RF) and (vii) maximum entropy (MAXENT). The models were calibrated with 70% of the data selected at random and the predictive performance of each model was evaluated on the remaining 30% [28] using the true skill statistic (TSS) [29]. This process was repeated 10 times (tenfold cross-validation), for each of the 10 background occurrence datasets, resulting in 700 models. An ensemble model was created using only models with TSS scores > 0.7 (Additional file 1: Fig. S4). This was then projected onto the whole world to assess suitability with the weighted mean method.

Human density maps for 2020 were downloaded at 30 arc seconds from the Center for International Earth Science Information Network [30]. Human density was then extracted for each occurrence of the native and invasive range of *L. fulica* and for the background occurrences. Only background occurrences that were within the area suitable for *L. fulica* were considered (based on the prediction of our ensemble model; Fig. 2A, B). As the data were non-normally distributed, human density differences between groups were determined using a Kruskal-Wallis test followed by a post hoc Dunn test for pairwise significance comparisons with Benjamini-Hochberg *p*-value adjustments [31].

### Global distribution of pet snails and pet owners’ behaviour

To assess the ownership of *L. fulica* as an exotic pet worldwide, we searched Instagram for posts referencing *L. fulica*. Instagram is a popular social media platform for sharing pictures and text about exotic pets, regroups over 1.4 billion active users worldwide (as of 2022; statista.com) and is reliable for monitoring emerging pet trades [32]. We collected all Instagram posts containing the hashtag *#achatinafulica*. We chose this keyword because it is among the most frequently used hashtags for referencing *L. fulica* on Instagram (based on a preliminary manual search), it is specific (contrarily to #achatina), and it is Latin and thus independent from the user’ language and geographical origin (contrarily to, for example, *#giantafricanlandsnails*). Our data mining campaign was carefully designed to not overload Instagram servers (i.e. several seconds separated each request). Only public data were retrieved, and all collected posts were anonymized [33, 34]. On November 2, 2022, we retrieved the text content (comments and responses) and geolocation (when available) of 30,039 unique posts (published since 2015) from 6640 unique Instagram users. Instagram users with no or more than one geolocation information were discarded and, among the 1667 remaining users, those using combinations of multiple non-specific and highly popular hashtags (e.g. *#love*, *#photography*, *#nature*, *#animals*, *#snail*, *#aquarium*) were removed (526 additional users removed). Most of these Instagram users corresponded to artificially grown accounts that use generic pictures and hashtags to build a follower base, probably with the objective to sell the account for targeted advertisement or to create revenue with sponsored content [35]. Then, we visited at least one random post from each of the 1141 remaining users and visually screened the picture(s) and comments to assess whether the user was really posting about *L. fulica*, whether the snail was depicted as a pet, an invasive species or a food resource, and whether there was a direct contact between the snail(s) and people skin (hand/body or face). Finally, to be able to compare the number of pet snail owners among countries, we divided the number of Instagram pet snail owners per country by the total number of Instagram users per country (obtained from napoleoncat.com).

## Results and discussion

### Pathogens carried by giant land snails and their potential mammalian hosts

We found that, over the last 60 years, 36 pathogen species have been documented to infect *L. fulica* (based on 73 scientific publications). However, the majority of these pathogens (80%) were found in *L. fulica* during the last 10 years, when the number of publications on pathogens of this species started to grow exponentially (Fig. 1A). It is therefore likely that many pathogens associated with giant land snails are yet to be discovered. Pathogen species found currently include 22 helminths, 7 bacteria and 7 protozoa. Among these 36 pathogens, 23 were identified to the species level (Fig. 1B). Most publications focused on a few pathogen species: the rat lungworm *Angiostrongylus cantonensis* (53/73 papers), the cat lungworm *Aelurostrongylus abstrusus* (7/73), *Angiostrongylus costaricensis* (4/53) and the French heartworm *Angiostrongylus vasorum* (4/53). All other pathogens were cited by just one or two papers (Table S2). The pathogen identified by our literature search had a wide variety of mammal hosts and could infect most domesticated mammal species, including household pets and livestock (Fig. 1C, D). Moreover, 15 of the pathogens recorded can infect humans (Fig. 1D). As most zoonotic pathogens carried by *L. fulica* can also infect domesticated mammals, popular household pets such as dogs and cats could serve as sentinel hosts for detecting pathogens transmitted by giant land snail and allow early detection of potential disease outbreaks [9, 36].

The rat lungworm, *A. cantonensis*, was the most frequently documented pathogen of *L. fulica*. This parasitic nematode causes eosinophilic meningitis in humans, a condition associated with severe neurological impairments in adults and death in young children [9, 37]. The rat lungworm is particularly concerning as, in countries invaded by *L. fulica*, it often infects > 20% of snails [9]. This parasitic nematode probably originates from Southeast Asia but it was reported all over the globe in the last century [9]. As a frequent intermediate host, *L. fulica* might facilitate the global spread of *A. cantonensis*, and other pathogens, at regional to global scale [8].

### Environmental suitability for giant land snails

Ensemble modelling based on bioclimatic data revealed high climatic suitability for *L. fulica* throughout all tropical regions. This suggests that the potential range of *L. fulica* is even larger than what is currently observed and that regions such as Northern Australia and Southern Japan could be invaded if the snail were accidentally or deliberately introduced (Fig. S3, Fig. 2A). Models’ performance was high with TSS between 0.88 and 0.99 for individual models (Additional file 1: Fig. S4) and equal to 0.87 for the final model. Explanative importance varied greatly among the five PCA axes used for modelling *L. fulica*’s climatic suitability, with PCA first axis being the most important (PCA axis: mean ± SD variable importance; PCA1: 0.9 ± 0.13; PCA2: 0.09 ± 0.06; PCA3: 0.06 ± 0.06; PCA4: 0.03 ± 0.02; PCA5: 0.1 ± 0.07).

Furthermore, inside the climatically suitable area for *L. fulica*, human population density differed between background occurrences and native and invasive *L. fulica* occurrences (Fig. 2B, Kruskal-Wallis test, *χ*^2^ = 1150.9, df = 2, *P* < 0.001). Pairwise comparisons using Dunn's test indicated that current invaded locations had the same human density as native occurrences (*P* > 0.05) and both the invasive (*P* < 0.001) and native (*P* < 0.001) occurrences were observed at higher human densities than background occurrences, indicating that giant land snails thrive in densely populated areas (Fig. 2B). This is likely to increase the opportunities for pathogen transmission to humans by multiplying direct contacts and the contamination of foodstuffs [38]. The risk of infection is especially high for young children that are more likely to put contaminated fingers, soil, objects or snails into their mouths [37]. Pathogen transmission from giant snails to humans can also occur by the direct consumption of undercooked snails [9]. Giant land snails are a culinary ingredient in many tropical regions and their presence near human settlements might encourage their consumption as an abundant and cheap food resource [9].

### Global distribution of pet snails and pet owners’ behaviour

In addition to being an edible invader in tropical regions, our social media survey revealed that giant land snails are popular exotic pets in Europe (Fig. 2C). We retrieved a total of 30,039 unique posts from 6640 unique Instagram users. These numbers are similar to the number of posts and Instagram users retrieved for the global pet trade in ants [32], suggesting that *L. fulica* alone is as popular as an entire emerging pet taxa. Among the 1141 users with geolocation information that we manually checked, 750 were really posting about *L. fulica*, including 623 depicting them as pets (Fig. 2C, Additional file 1: Fig.S5), 114 as invasive species and 13 as a food resource (including 10 from Nigeria) (Additional file 1: Fig. S6). As temperate climates are unsuitable for *L. fulica*, the pet trade poses a low risk of further spread and establishment of populations at outdoor locations in Europe (Fig. 2A); however, pathogen transmission from individual pets is still an important risk. Our social media survey showed that pet snail keepers commonly hold their snails in their hands and occasionally on their face (Fig. 1D), a behaviour likely to greatly favour pathogen transmission between snails and humans. This suggests that pet owners are not aware of the health risks associated with giant land snails. These risks seem to have been overlooked so far, given that we identified only a single study that screened pathogens of pet snails [39], detecting four different nematode species in 60 *L. fulica* individuals from three private collections in Italy (but not the rat lungworm). Our findings highlight the usefulness of social media data for investigating potential threats associated with exotic pets. However, a more comprehensive sampling design that included additional search terms, languages or social media platforms would allow a more exhaustive risk assessment. For example, we may have missed some countries where Instagram is unpopular or unavailable (typically China) and some age groups, as > 60% of Instagram users are aged between 18 and 34 (statista.com), which may not represent the full spectrum of exotic pet owners or the average risk behaviour of pet owners.

## Conclusions

Biological invasions and emerging pet trades will continue to grow in the coming decades [40, 41]. Unavoidably, this will create more opportunities for the introduction and spread of harmful pathogens to humans and other animals [8, 42]. Our results highlight the importance of accounting for multiple types of human-wildlife interactions when assessing risks of infectious disease emergence. Furthermore, by targeting the species most likely to spread pathogens, we show that it is possible to rapidly identify emerging disease risks on a global scale, thus guiding timely and appropriate responses.

### Supplementary Information

**Additional file 1: Fig. S1.** First five axes of the principal component analysis (PCA) computed with the 19 bioclimatic variables. **Fig. S2.** Standardized scores of the 19 bioclimatic variables on the five PCA axes used to model global climatic suitability for *Lissachatina fulica*. **Fig. S3.** Occurrence points used to calibrate and validate species distribution models. **A** Native (green) and invasive (purple) GBIF occurrences of *Lissachatina fulica* (after cleaning and spatial thinning). **B** Background dataset containing 115,162 GBIF occurrences (after cleaning and spatial thinning) of 3848 terrestrial gastropod species in the order Stylommatophora. Only a random subset of 10,000 occurrences is displayed here. **Fig. S4.** True skill statistics (TSS) scores for each algorithm used in the ensemble model of *Lissachatina fulica* climatic suitability: generalized linear model (GLM), generalized boosting model (GBM), classification tree analysis (CTA), artificial neural networks (ANN), multiple adaptive regression splines (MARS), random forest (RF) and maximum entropy (MAXENT). **Fig. S5.** Number of Instagram users referencing *Lissachatina fulica* as a pet per country (countries with only one user were not displayed). **Fig. S6.** Number of Instagram users referencing *Lissachatina fulica* as an invasive species (yellow bars) or as a food resource (orange bar) per country (countries with only one user were not displayed). **Dataset S1** (separate file). List of articles reviewed for evaluating the number and identity of pathogens carried by the giant land snail *Lissachatina fulica*. **Dataset S2** (separate file). List of pathogens carried by carried by the giant land snail *Lissachatina fulica*. **Dataset S3** (separate file). Host-pathogen associations for the 25 pathogens of *Lissachatina fulica* identified at the species level. **Dataset S4** (separate file). R files allowing replication of the ensemble model performed to predict environmental suitability for *Lissachatina fulica*. This .Rdata object contains the cleaned and thinned GBIF occurrences for *L. fulica* presence and background (i.e. occurrences of Stylommatophora mollusks); the R script necessary to prepare data and run models; the R script necessary to prepare data and test differences in human density between *L. fulica* occurrences (native and invasive) and background occurrences. **Dataset S5** (separate file). Number of Instagram users referencing *Lissachatina fulica* as pets per country and total number of Instagram users per country (source: napoleoncat.com).

## Data Availability

The complete data file and methods are publicly available in the public GitHub repository: https://github.com/JGippet/Gippet2023_GiantLandSnails
