# Supplementary material for: The global risk of infectious disease emergence from giant land snail invasion and pet trade
Source: Parasit Vectors. 2023 Oct 17;16:363. doi: 10.1186/s13071-023-06000-y (PMC10580515; doi:10.1186/s13071-023-06000-y)
Supplement: Supplementary file 1 — Additional file 1: Fig. S1. First five axes of the principal component analysis (PCA) computed with the 19 bioclimatic variables. Fig. S2. Standardized scores of the 19 bioclimatic variables on the five PCA axes used to model global climatic suitability for Lissachatina fulica. Fig. S3. Occurrence points used to calibrate and validate species distribution models. A Native (green) and invasive (purple) GBIF occurrences of Lissachatina fulica (after cleaning and spatial thinning). B Background dataset containing 115,162 GBIF occurrences (after cleaning and spatial thinning) of 3848 terrestrial gastropod species in the order Stylommatophora. Only a random subset of 10,000 occurrences is displayed here. Fig. S4. True skill statistics (TSS) scores for each algorithm used in the ensemble model of Lissachatina fulica climatic suitability: generalized linear model (GLM), generalized boosting model (GBM), classification tree analysis (CTA), artificial neural networks (ANN), multiple adaptive regression splines (MARS), random forest (RF) and maximum entropy (MAXENT). Fig. S5. Number of Instagram users referencing Lissachatina fulica as a pet per country (countries with only one user were not displayed). Fig. S6. Number of Instagram users referencing Lissachatina fulica as an invasive species (yellow bars) or as a food resource (orange bar) per country (countries with only one user were not displayed). Dataset S1 (separate file). List of articles reviewed for evaluating the number and identity of pathogens carried by the giant land snail Lissachatina fulica. Dataset S2 (separate file). List of pathogens carried by carried by the giant land snail Lissachatina fulica. Dataset S3 (separate file). Host-pathogen associations for the 25 pathogens of Lissachatina fulica identified at the species level. Dataset S4 (separate file). R files allowing replication of the ensemble model performed to predict environmental suitability for Lissachatina fulica. This .Rdata object contains the c [file 13071_2023_6000_MOESM1_ESM.docx]

**Additional file for**

The global risk of infectious disease emergence from giant land snail invasion and pet trade

**This PDF file includes:**

Figures S1 to S6

Legends for Datasets S1 to S5

**Other supporting materials for this manuscript include the following:**

Datasets S1 to S5 (Access at: XXXX available upon publication)


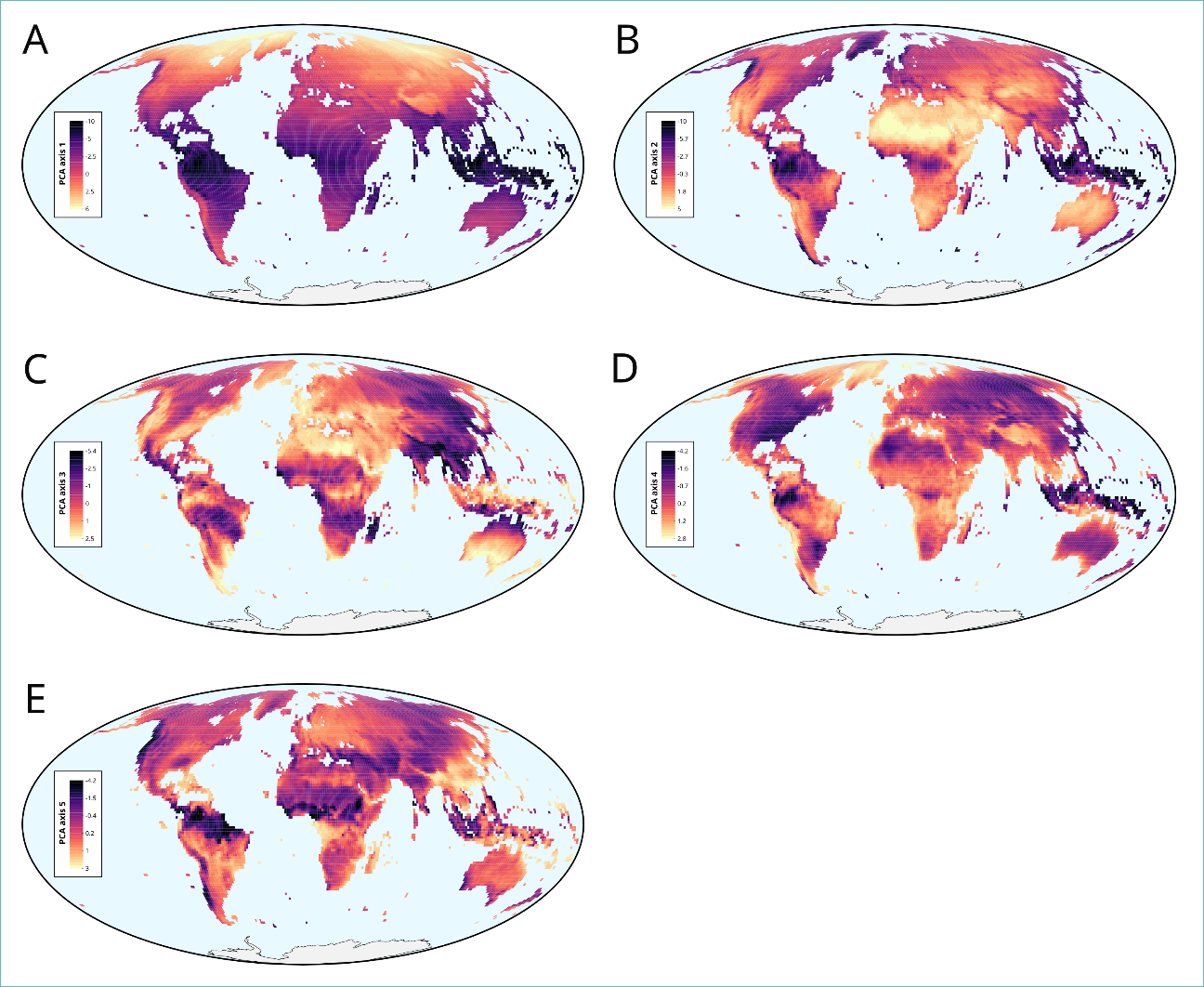


**Fig. S1:** First 5 axes of the Principal Component Analysis (PCA) computed with the 19 bioclimatic variables.


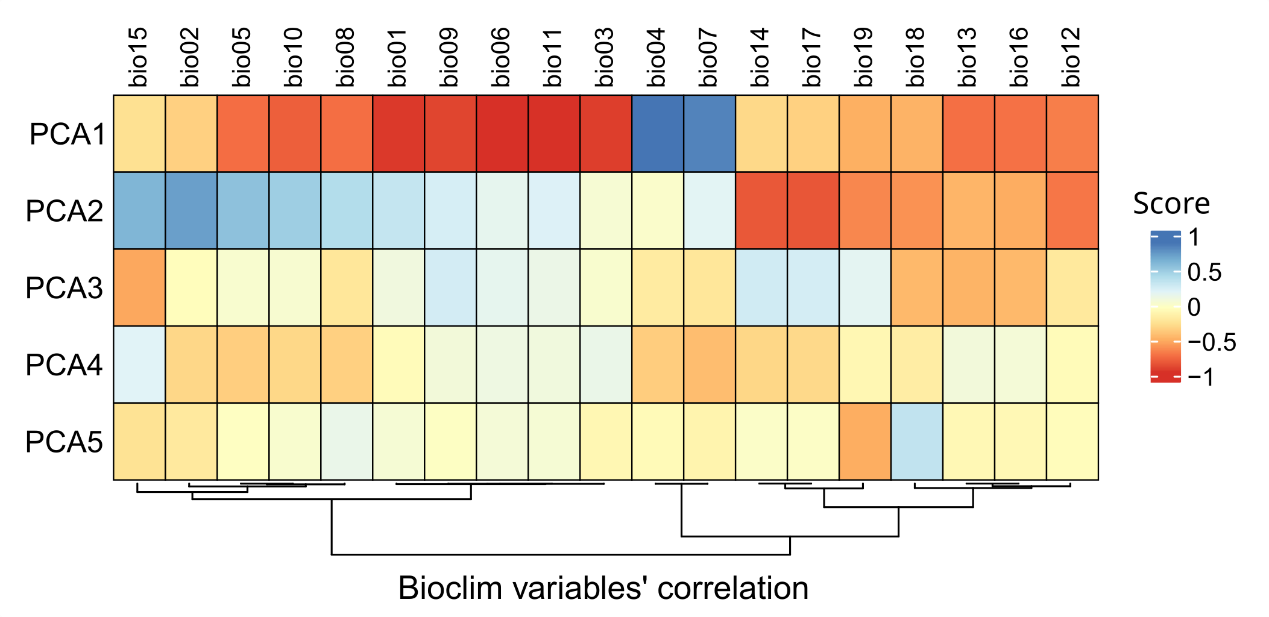
**Fig. S2:** Standardized scores of the 19 bioclimatic variables on the five PCA axes used to model global climatic suitability for *L. fulica*.


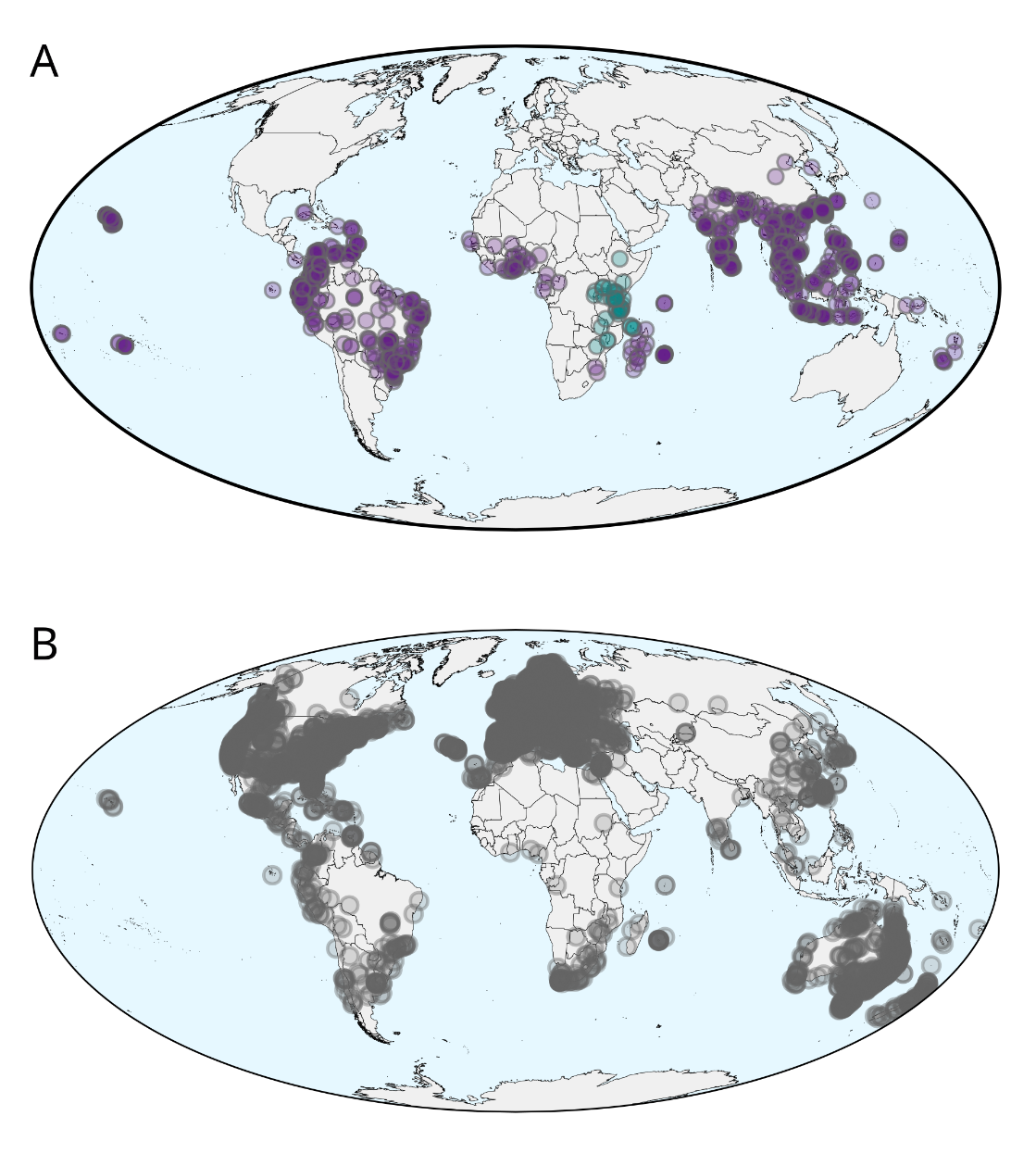


**Fig. S3:** Occurrence points used to calibrate and validate species distribution models. **(A)** Native (green) and invasive (purple) GBIF occurrences of *L. fulica* (after cleaning and spatial thinning). **(B)** Background dataset containing 115,162 GBIF occurrences (after cleaning and spatial thinning) of 3,848 terrestrial gastropod species in the order Stylommatophora. Only a random subset of 10,000 occurrences is displayed here.


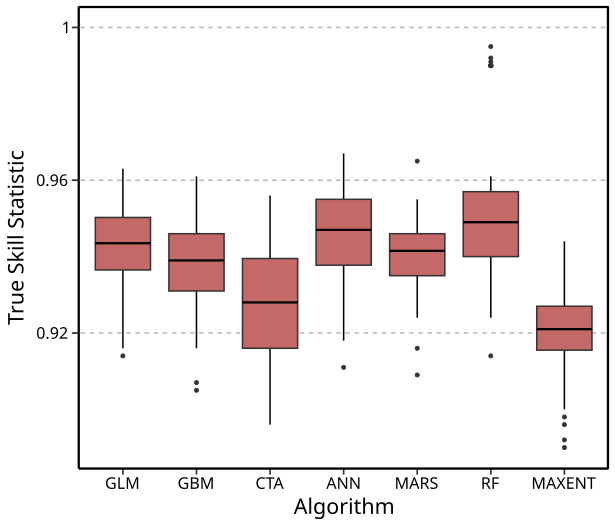


**Fig. S4:** True skill statistics (TSS) scores for each algorithm used in the ensemble model of *L. fulica* climatic suitability: Generalized linear model (GLM), Generalized boosting model (GBM), Classification tree analysis (CTA), Artificial neural networks (ANN), Multiple adaptive regression splines (MARS), random forest (RF) and maximum entropy (MAXENT).


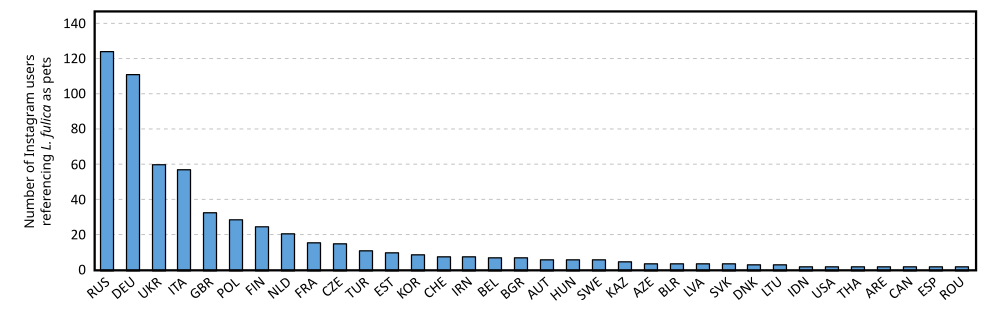
**Fig. S5:** Number of Instagram users referencing *L. fulica* as a pet per country (countries with only one user were not displayed).


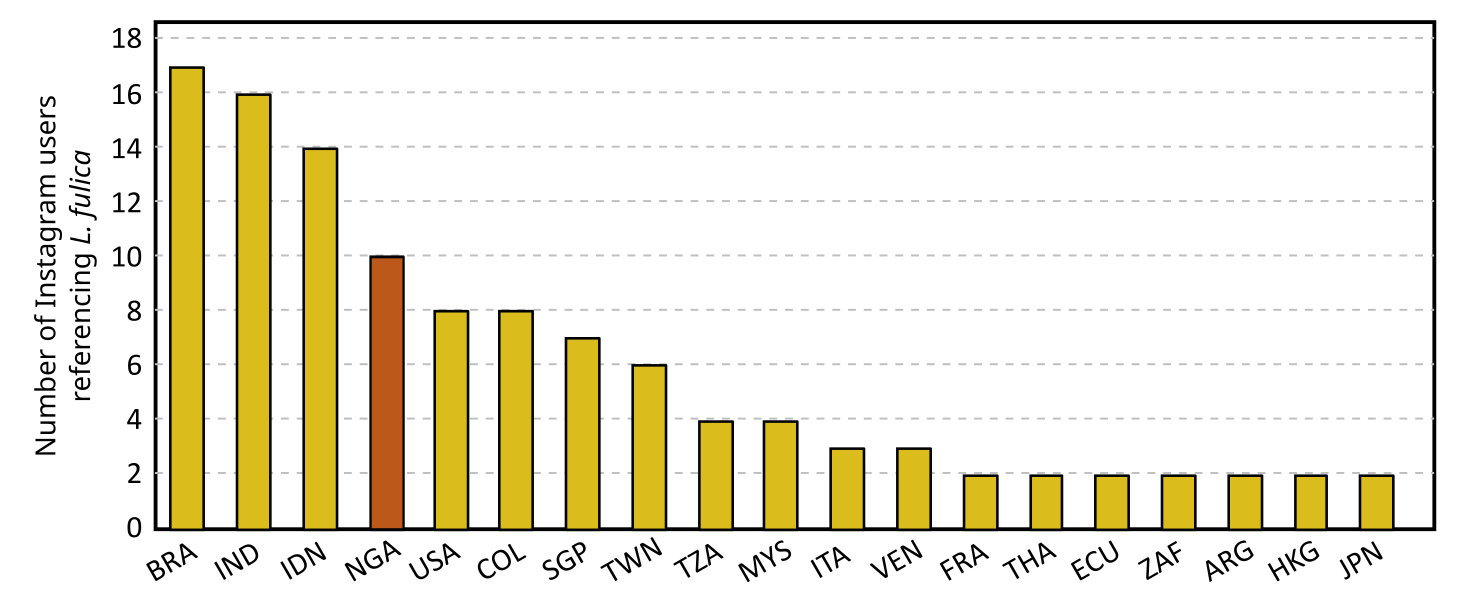


**Fig. S6:** Number of Instagram users referencing *L. fulica* as an invasive species (yellow bars) or as a food resource (orange bar) per country (countries with only one user were not displayed).

Dataset S1 (separate file). List of articles reviewed for evaluating the number and identity of pathogens carried by the giant land snail *Lissachatina fulica*.

Dataset S2 (separate file). List of pathogens carried by carried by the giant land snail *L. fulica*.

Dataset S3 (separate file). Hosts-pathogens associations for the 25 pathogens of *L. fulica* identified at the species level.

Dataset S4 (separate file). R files allowing replication of the ensemble model performed to predict environmental suitability for *L. fulica*. This .Rdata object contains the cleaned and thinned GBIF occurrences for *L. fulica* presence and background (i.e., occurrences of Stylommatophora mollusks); the R script necessary to prepare data and run models; the R script necessary to prepare data and test differences in human density between *L. fulica* occurrences (native and invasive) and background occurrences.

Dataset S5 (separate file). Number of Instagram users referencing *L. fulica* as pets per country and total number of Instagram users per country (source: napoleoncat.com).
